# Q&A: Antibiotic resistance: what more do we know and what more can we do?

**DOI:** 10.1186/1741-7007-11-51

**Published:** 2013-05-17

**Authors:** Gerard D Wright

**Affiliations:** 1 Michael G DeGroote Institute for Infectious Disease Research and the Department of Biochemistry and Biomedical Sciences, McMaster University, 1280 Main St W., Hamilton, L8S 4K1, Canada

## Is the problem of antibiotic resistance getting worse?

Yes. Resistance to antibiotics continues to be a significant and growing medical problem across the globe. In the US, the Centers for Disease Control recently released a report showing that infections due to carbapenem-resistant Enterobacteriaceae (CRE), which are associated with mortality rates between 40% and 50%, rose from 1.2% to 4.2% over the decade from 2001 to 2011 [1]. In the clinically important *Klebsiella* subset of these pathogens, the rise over the same time period was from 1.6% to 10.4%. Carbapenems are among the last resort antibiotics we have to treat infections of Gram-negative bacteria and this steady erosion of their efficacy is especially concerning. The cause is the spread of genes that encode enzymes that destroy these antibiotics, in particular KPC (*Klebsiella pneumoniae* carbapenemase) and NDM (New Delhi metallo-beta-lactamase) [2]. The latter has been found widespread in the environment, including the water supply on the Indian subcontinent [3].

Infections due to multidrug resistant *Neisseria gonorrhea* are also on the rise. Once easily treated with available antibiotics, the emergence of drug-resistant strains resulting in clinical failures is becoming more common. A recent study in a Toronto clinic showed that 6.77% of cases could not be cured with standard oral antibiotic therapy [4]. Outbreaks of infections caused by multidrug and sometimes pan-resistant epidemic clones of *Acinetobacter baumannii* are increasingly reported in health care settings across the globe. The establishment of methicillin-resistant *Staphylococcus aureus* (MRSA) in the community at large as well as in hospitals is continuing; over 460,000 MRSA infections required hospitalization in the US in 2009 [5]. There are now circulating strains of the tuberculosis-causing bacillus *Mycobacterium tuberculosis* that are totally drug resistant (TDR) [6]. And the list goes on…

The result of these trends has been increased attention and alarm by clinicians and the public health community. Sally Davies, the Chief Medical Officer of the UK, recently called the situation apocalyptic and ranked antibiotic resistance as a threat as important as terrorism. The WHO World Health Day on 7 April 2011 was focused on antibiotic resistance with the tag line ‘Antibiotic resistance: no action today, no cure tomorrow’. The head of the US CDC Thomas Frieden called antibiotic resistance a ‘nightmare’.

## Is all this hype or is the problem that bad?

It is important to realize that antibiotic resistance is a natural phenomenon. It is the result of selection for genetic elements in bacteria that confer the ability to continue to grow in the presence of otherwise toxic compounds. This evolutionary process has been going on for as long as bacteria have had to cope with toxic molecules; in other words, for millennia. Evidence of this can be found in studies that show that resistance genes are prevalent in 30,000-year-old permafrost samples, and in bacteria living in a cave, sealed from the surface 4 million years ago [7,8]. Furthermore, the ability of bacteria to exchange these genes through mobile genetic elements such as plasmids ensures that antibiotic resistance traits can be spread efficiently through bacterial communities. Recently, it has been confirmed that the antibiotic resistance genes in environmental bacteria are the same ones found in pathogens [9]. The prevalence of global travel means that microbes and their resistance genes can move with unprecedented ease. Resistance, therefore, is ancient and widespread.

These facts conspire to make antibiotics very unusual drugs in that their use selects for their eventual obsolescence. We will therefore always be in need of new drugs to fill the ‘pipeline’. The problem we have now is that this pipeline is pretty much dry. There are few new antibiotics coming to market or in clinical trials, certainly not enough to address the resistance issue over the long term. Since it can take up to a decade to get a new drug from the lab to the pharmacy, we are not in a good place right now and the problem will continue to get worse.

## Why is there a shortage of new antibiotic drugs?

The pharmaceutical industry has been responsible for bringing all the antibiotics in current clinical use to market over the past 70 years. They have been remarkably good at finding new molecules, either completely synthetic ones or more often natural products made by fungi and bacteria themselves, that serve as starting points for antibiotic drugs. By carefully building on these chemical scaffolds, the pharmaceutical industry has provided us with a plentiful supply of safe and effective antibiotics that can act on most pathogens, for more than 70 years. It may actually be this success, however, that is impeding new drug discovery in this field now.

First, most of the blockbuster successful antibiotics brought to market over the past decades are off-patent, and as a result, are very inexpensive. This is good news for cash-strapped health care providers and patients, but bad news for pharmaceutical research that needs profits to invest in fundamental discoveries that will result in the next new drugs. Furthermore, while resistance is growing and very serious, many of these off-patent drugs continue to be effective in the majority of cases. Faced with using an expensive new drug or a cheaper old one, many clinicians opt for the less expensive option. Furthermore, if clinicians feel that a new antibiotic should be used only sparingly to forestall resistance, then the new drug will not be used as much. The paradoxical result is a contracting market, and a concern on the part of the company that they will not make sufficient profit during the lifetime of the patent exclusivity to recoup their investments and fuel new discovery.

Second, drugs today are very safe. This is the result of the vigilance of government agencies, such as the FDA in the US, that set the rules for drug clinical trials. In these trials, the safety and efficacy of new drugs is assessed over several sites, and often several years, and the results adjudicated by an independent panel of experts who decide if the experimental drug candidate has met the criteria to be a new marketable drug. Any new drug candidate must be rigorously proven to be safe. The current safety standards are highly stringent and it has been pointed out that our old antibiotics such as penicillin, which has proven to be a lifesaver on a global scale, would in all likelihood not pass current safety muster. Furthermore, new antibiotics that target drug-resistant pathogens have some specific disadvantages under the current regulatory system. For obvious ethical reasons, new candidate antibiotics cannot be compared with a placebo but instead must be compared with an effective drug already on the market. Usually patients cannot be pre-screened to determine whether they are infected with an antibiotic-resistant or sensitive organism. If resistant organisms are not highly prevalent in the specific hospital site where the trial is being conducted, the new compound may not show statistical efficacy compared with an older one. The result then, is a requirement to enlist very large numbers of patients to show statistical efficacy of the new drug; this is incredibly expensive and time-consuming.

Third, infections that require antibiotics very often present acutely to the clinician with rather ambiguous symptoms (fever, inflammation, and so on). Physicians, therefore, treat patients empirically without knowing the actual causative pathogen since it is often impossible to wait 24 to 48 hours for traditional culture-based diagnostic methods, which themselves are problematic as the causative agents are often not readily cultured. This empirical strategy results in a desire for broad-spectrum antibiotics, drugs that are effective against many pathogens. Broad-spectrum agents are also in the company’s interest as it means that their drug will be prescribed more, ensuring more revenue. As a result, broad-spectrum efficacy is generally one of the important criteria to be met in most antibiotic drug discovery campaigns, even though a good argument can be made that such agents promote resistance, and are associated with undesirable side effects, such as antibiotic-associated colitis, due to their indiscriminate effect on the patient’s microbiome. This requirement for broad-spectrum activity results in a significant triage of drug candidates early in the drug discovery process, as compounds with the desired traits have been shown to be quite rare.

Finally, unlike most other drugs, antibiotics are prescribed for a very short term and actually cure disease. Again, this is great news for the patient, but challenging for a company that desires a sufficient return on its investment. All of these issues conspire to make antibiotics less attractive as targets for the pharmaceutical industry in comparison to other therapeutic areas. As a result, many large pharmaceutical companies have either abandoned, or greatly reduced, their antibiotic discovery programs. This has had two direct effects. The first is a dry antibiotic drug pipeline and the second is a diaspora of antibiotic discovery expertise. Since these firms have either shut down antibiotic discovery groups or re-assigned individuals to other areas, the wealth of knowledge that was once embedded in these teams is vanishing. As a result, it will not be easy simply to ‘turn on the discovery tap’ even if economic and scientific challenges can be met to encourage new antibiotic drug development.

## Is there any hope?

Paradoxically, while there is great clinical need that will only get more acute given the inexorability of the evolution of drug resistance, these are grim times for the antibiotic drug discovery field [10]. The retreat of pharmaceutical companies from this area has a direct consequence: fewer new medicines to treat bacterial infections over the short and medium term. There are, however, some reasons to be hopeful. Bedaquiline (Sirturo), a first-in-class inhibitor of the *Mycobacterium tuberculosis* ATP synthase, received FDA approval in 2012 for the treatment of multi-drug-resistant tuberculosis. Fidaxomicin (Dificid) is a selective agent for *Clostridium difficile* that received FDA approval in 2011 and, similarly, surotomycin (Cubist) is a compound directed to *C. difficile* in phase III clinical trials. These examples demonstrate that narrow spectrum agents have their place in drug development and can be brought to market. New agents continue to be identified, such as Achaogen’s plazomicin, a semi-synthetic aminoglycoside antibiotic with broad spectrum activity that has just successfully completed phase II trials for urinary tract infections.

An alternative approach to new antibiotic drug discovery is the pairing of existing drugs with antibiotic adjuvants, compounds that increase the potency of antibiotic drugs. This approach includes the inhibition of resistance and at least two new combinations of beta-lactam drugs with inhibitors of inactivating beta-lactamases are in phase III clinical trials: ceftolozane/tazobactam (Cubist), and ceftazidime/avibactam (Astra-Zeneca, Forrest). This strategy of combining an antibiotic with other bioactive compounds to overcome resistance and improve efficacy can be extended to other compounds as well. For example, a mixture of cefuroxime and the anti-platelet drug ticlopidine (Ticlid) has unexpected and specific synergy against MRSA [11]. There is tremendous scope for further combinatorial innovation of this kind [12].

Finally, there is, in fact, no shortage of compounds with antibiotic activity. Natural products of microbial origin are particularly rich in such activities. The challenge with these compounds is that they are lousy drugs at present. The criteria of single agents with broad spectrum utility, minimal resistance, general availability (oral, intravenous and so on), and low cost that has driven antibiotic drug discovery over the past decades may, however, prove too strict for the future.

To reach a turning point where new antibiotic drug discovery will begin anew on the scale needed to meet the challenge of evolution and resistance will require new funding models. A good start to achieving this goal is the Generate Antibiotic Incentives Now (GAIN) Act passed by the US congress in 2012 [13]. It offers patent exclusivity extensions for new antibiotics active against resistant bacteria as well as measures to overcome clinical trial challenges, and to fast track new drugs. Indeed, some of the drug candidates mentioned above are benefiting already from this initiative.

Further measures to re-tool the clinical trials requirements for new antibiotics have recently been tabled by representatives of the pharmaceutical industry still active in antibiotics research [14]. In their proposal, the authors present a spectrum of options to reform the clinical trials guidelines to address the specific needs of antibiotics including a framework for predictive smaller and focused trials along with options for disease or pathogen-specific indications. These creative solutions offer a way forward through the current regulatory challenges that new antibiotics face.

In the meantime though, we are left with an increasingly bare antibiotic drug cupboard.
